# Robotic versus laparoscopic gastrectomy for gastric cancer: an umbrella review of systematic reviews and meta-analyses

**DOI:** 10.1007/s13304-021-01059-7

**Published:** 2021-05-25

**Authors:** Luigi Marano, Daniele Fusario, Vinno Savelli, Daniele Marrelli, Franco Roviello

**Affiliations:** Unit of General Surgery and Surgical Oncology, Department of Medicine, Surgery and Neurosciences, University of Siena, Strada delle Scotte, 4, 53100 Siena, Italy

**Keywords:** Adult surgery, Gastrointestinal tumours, Oncology, Systematic review, Robotic surgery

## Abstract

An umbrella review was performed to summarize literature data and to investigate benefits and harm of robotic gastrectomy (RG) compared to laparoscopic (LG) approach. To overcome the intrinsic limitations of laparoscopy, the robotic approach is claimed to facilitate lymph-node dissection and complex reconstruction after gastrectomy, to assure oncologic safety also in advanced gastric cancer. A literature search was conducted in PubMed, Cochrane and Embase databases for all meta-analyses published up to December 2019. The search strategy was previously published in a protocol. We selected fourteen meta-analyses comparing outcomes between LG and RG with curative intent in patients with diagnosis of resectable gastric cancer. We highlight that RG has a longer operation time, inferior blood loss, reduction in hospital stay and a more rapid recovery of bowel function. In meta-analyses with statistical significance the number of nodes removed in RG is higher than LG and the distal margin of resection is higher. There is no difference in terms of total complication rate, mortality, morbidity, anastomotic leakage, anastomotic stenosis, intestinal obstruction and in conversion rate to open technique. The safety and efficacy of robotic gastrectomy are not clearly supported by strong evidence, suggesting that the outcomes reported for each surgical technique need to be interpreted with caution, in particular for the meta-analyses in which the heterogeneity is large. Certainly, robotic gastrectomy is associated with shorter time to oral intake, lesser intraoperative bleeding and longer operation time with an acceptable level of evidence. On the other hand, the data regarding other outcomes are insufficient as well as non-significant, from an evidence point of view, to draw any robust conclusion.

## Introduction

Gastric cancer (GC) is the fifth most common malignant neoplasm and the third leading cause of cancer related deaths globally and for resectable gastric cancer (GC) patients the recommended surgical procedure is the standard gastrectomy with D2 lymphadenectomy [1]. the Japanese, Korean, Italian, German and British national guidelines recommend D2 procedure as the standard of surgical treatment with curative intent, as reported by the European Society for Medical Oncology (ESMO) guidelines, as well as the joint ESMO—European Society of Surgical Oncology (ESSO)—European Society of Radiotherapy and Oncology (ESTRO) guidelines [2].

During the last decades, minimally invasive surgery of the stomach has become increasingly employed worldwide. Laparoscopic gastrectomy (LG) has been routinely used for the treatment of GC, supported by strong evidence that LG is technically safe and leads to better short-term outcomes than conventional open gastrectomy for early stage gastric cancer [3–12]. However, diffusion of laparoscopic surgery is limited by technical difficulties regarding total gastrectomy procedure as well as D2 lymphadenectomy, that entails the removal of node stations along the celiac trunk, left gastric artery and hepatic pedicle [13, 14]; these factors limit the execution of a correct D2 spleen-preserving laparoscopic gastrectomy (LG) for the treatment of advanced gastric cancer only to high-volume centres.

Since the first robot-assisted gastrectomy reported by Hashizume et al. in 2003 [15], robotic gastrectomy (RG) is claimed to facilitate complex reconstruction after gastrectomy and lymph node dissection, to assure oncologic safety also in advanced gastric cancer patients [16–18]. In current literature, many observational studies reported the effectiveness and safety of RG [19–23] and previous meta-analyses [24–26] highlighted a lower complication rate as well as bleeding in the robotic approach group when compared to the laparoscopic one.

Since several previous systematic reviews on the comparison of RG and LG are available and timely evidence is required to inform the scientific community, we believed a de novo systematic review was inappropriate, and, as reported in our published protocol [27], we performed a comprehensive umbrella review to collect and assess information from previous systematic reviews that have compared the laparoscopic with robotic gastrectomy.

Umbrella reviews are syntheses of existing systematic reviews and/or meta-analyses providing an ideal method to comprehensively review the evidence base and to explore the contradictory findings of previous reviews [28].

The aim of this review is to investigate the benefits and harm of robotic gastrectomy compared with laparoscopic approach searching between the findings of high-quality systematic reviews, to give surgeons and policymakers a comprehensive overview of the depth and strength of the scientific evidence to evaluate the feasibility of the robotic gastrectomy for gastric cancer.

## Methods and analysis

This umbrella review was designed using the methodology guidelines for umbrella reviews provided by the Joanna Briggs Institute [28]. As well, we followed the Preferred Reporting Items for Systematic review and Meta-Analyses (PRISMA) guidelines [29] (Appendix 1). The protocol has been registered with PROSPERO (no. CRD42019139906) and has been published [27]. The review was performed following the protocol without deviation.

### Search strategy, study selection and data collection

We searched for systematic review and meta-analysis comparing the outcomes of robotic gastrectomy (RG) and laparoscopic gastrectomy (LG) in patients with gastric cancer. A literature search was conducted in PubMed, Cochrane and Embase databases for all articles published up to December 2019. The “related article” function from PubMed will be used to further identify potential articles that were eligible for inclusion in the review. The bibliography of all selected articles will be hand searched to identify additional articles that met our inclusion criteria [27].

Two independent reviewers (LM and DF) had screen titles, abstract and full-text records in duplicate. Data were extracted by two authors (LM and DF), who independently reviewed and screened all eligible studies for content according to the inclusion criteria indicated in the protocol. We extracted only data pertaining to the comparison between RG and LG. The quality of the included studies was assessed using the appropriate AMSTAR (A Measurement Tool to Assess Systematic Reviews) [30] checklist by the two reviewers: of the included studies only one had scored 6 points on the AMSTAR check list, the others had scored 7 or more points (Table 1).

**Table 1:** AMSTAR score for included meta-analyses

Studies	Uses elements of PICO	A priori research design	Explained selection of the study designs	Comprehensive literature search	Study selection in duplicate	Dual data extraction	Excluded study list provided	Included studies described	Funding sources reported	Quality assessed	Quality used appropriately	Satisfactory discussion of heterogeneity	Conflicts of interest reported	AMSTAR2
[33]	**✔**	**–**	**✔**	**✔**	**✔**	**✔**	**–**	**X**	**–**	**✔**	**✔**	**✔**	**✔ˣ**	**9**
[38]	**✔**	**–**	**✔**	**✔**	**–**	**✔**	**X**	**X**	**–**	**✔**	**✔**	**✔**	**✔**^a^	**8**
[39]	**✔**	**–**	**–**	**✔**	**✔**	**✔**	**X**	**X**	**–**	**✔**	**✔**	**✔**	**✔ˣ**	**8**
[40]	**✔**	**–**	**✔**	**✔**	**✔**	**✔**	**✔**	**X**	**–**	**✔**	**✔**	**✔**	**✔**^a^	**10**
[41]	**✔**	**–**	**✔**	**✔**	**–**	**✔**	**X**	**X**	**–**	**✔**	**✔**	**✔**	**✔ˣ**	**8**
[42]	**✔**	**–**	**✔**	**✔**	**–**	**✔**	**X**	**X**	**–**	**✔**	**✔**	**–**	**✔ˣ**	**7**
[43]	**✔**	**–**	**✔**	**✔**	**–**	**✔**	**✔**	**X**	**–**	**✔**	**✔**	**–**	**✔ˣ**	**8**
[44]	**✔**	**–**	**✔**	**✔**	**✔**	**✔**	**X**	**X**	**–**	**✔**	**✔**	**–**	**✔ˣ**	**8**
[36]	**✔**	**–**	**✔**	**✔**	**✔**	**✔**	**X**	**X**	**–**	**✔**	**✔**	**✔**	**✔ˣ**	**9**
[23]	**✔**	**–**	**✔**	**✔**	**–**	**✔**	**X**	**X**	**–**	**✔**	**✔**	**✔**	**–**	**7**
[34]	**✔**	**–**	**✔**	**✔**	**✔**	**✔**	**X**	**X**	**–**	**✔**	**✔**	**✔**	**–**	**8**
[35]	**✔**	**–**	**✔**	**✔**	**–**	**✔**	**X**	**X**	**–**	**✔**	**✔**	**✔**	**✔ˣ**	**8**
[36]	**✔**	**–**	**✔**	**✔**	**–**	**–**	**X**	**X**	**–**	**✔**	**✔**	**✔**	**–**	**6**
[37]	**✔**	**–**	**✔**	**✔**	**✔**	**✔**	**X**	**X**	**–**	**✔**	**✔**	**✔**	**✔ˣ**	**9**

x = Partial Yes **✔ = **Yes –**= **No

^a^The authors described their funding sources and how they managed potential conflicts of interest

ˣThe authors declare no conflict of interest

### Statistical analyses

For each meta-analysis, we estimated the summary effect size and its 95% CI using random-effects models. For the largest study of each meta-analysis, we estimated the SE of the effect size and we examined whether the SE was less than 0.1. In a study with SE of less than 0.1, the difference between the effect estimate and the upper or lower 95% confidence interval is less than 0.2 (i.e., this uncertainty is less than what is considered a small effect size). In case of meta-analyses with continuous data, the effect estimate was transformed to an odds ratio with an established formula [31]. Between-study heterogeneity was assessed via the *I*^2^ metric. *I*^2^ ranges between 0 and 100% and is the ratio of between-study variance over the sum of the within- and between-study variances. Values exceeding 50% are usually considered to represent large heterogeneity.

We evaluated whether there was evidence for small-study effects using the Egger p test [32] A P value less than 0.1 with more conservative effect in larger studies judged to be evidence for small-study effects. We applied the excess statistical significance test, which evaluates whether the observed number of studies with nominally statistically significant results.

Finally, we identified outcomes that had the strongest statistical support for association and no signals of high heterogeneity or bias. Specifically, we used the following categories:

- convincing (class I) when number of cases > 1000, *p* < 10 − 6, I2 < 50%, 95% prediction interval excluding the null, no small-study effects and no excess significance bias:
- highly suggestive (class II) when number of cases > 1000, *p* < 10 − 6, largest study with a statistically significant effect and class I criteria not met;
- suggestive (class III) when number of cases > 1000, *p* < 10 − 3 and class I–II criteria not met;
- weak (class IV) when *p* < 0.05 and class I–III criteria not met;
- non-significant when *p* > 0.05.

The statistical analysis and the power calculations were done with STATA version 12.0.

## Results

### Search strategy

One hundred and fifty-six records were found from our literature search. (Appendix 2). Of these, 137 were excluded after a rapid screening of title and abstract. The other five articles were excluded after full-text screening. In total, we selected 14 meta-analyses (Table 2) The full list of the included studies is available in Appendix 3.

**Table 2:** Studies characteristic

Studies	Design	Continent (if specificated country)		N. participants	RG	LG	N. studies
[39]	Meta-analysis	Asia	Europe (Italy)	918	268	650	3
[38]	Meta-analysis	Asia	Europe (Italy)	2235	762	1473	7
[39]	Meta-analysis	Asia	Europe (Italy)	2495	736	1759	9
[40]	Meta-analysis	Asia	Europe (Italy)	1249	404	845	6
[41]	Meta-analysis	Asia	Europe (Italy)	1870	634	1236	8
[42]	Meta-analysis	Asia	Europe (Italy)	1875	506	1369	8
[43]	Meta-analysis	Asia	Europe (Italy)	3204	997	2207	11
[44]	Meta-analysis	Asia		1796	551	1245	5
[36]	Meta-analysis	Asia		562	165	397	3
[23]	Meta-analysis	Asia		3580	1096	2484	12
[34]	Meta-analysis	Asia	Europe (Italy)	3503	993	2510	11
[35]	Meta-analysis	Asia	Europe (Italy)	5953	1830	4123	19
[36]	Meta-analysis	Asia	Europe (Italy)	3744	1134	2610	12
[37]	Meta-analysis	Asia	Europe (Italy)	4576	1517	3059	16

Green are the meta-analyses that compares RG to LG and to Open Gastrectomy

### Review characteristics

All the 14 included meta-analyses [23, 33–45] compare short-term outcomes between laparoscopic and robotic total/subtotal gastrectomy with curative intent in adult patients with diagnosis of resectable gastric cancer (Table 3).

**Table 3:** Visive indicator of the outcomes

Meta-analysis	Time	Blod loss	Conversion rate	Harvested lymphnodes	Hospital stay	Mortality rate	Morbidity	Total complication	Anastomotic leakage	Anastomotic stenosis	Intestinal obstruction	Proximal margin	Distal margin	Time to first flatus	Oral intake
[33]	X	X		X	X	X	X								
[38]	X	X		X	X	X		X	X	X	X	X	X		
[39]	X	X	X	X	X	X	X		X	X	X	X	X	X	X
[40]	X	X		X	X			X							
[41]	X	X		X	X			X	X	X		X	X		
[42]	X	X		X	X			X				X	X		
[43]	X	X		X	X	X	X		X						
[44]	X	X		X	X			X	X			X	X		
[36]	X	X		X	X			X							
[23]	X	X	X	X	X	X		X				X	X	X	
[34]	X	X		X	X			X						X	X
[35]	X	X		X	X	X		X				X	X	X	X
[36]	X	X		X	X			X				X	X		
[37]	X	X	X	X	X	X		X				X	X	X	
TOT	14	14	3	14	14	7	3	11	5	3	2	9	9	5	3

Green are the meta-analyses that compares RG to LG and to Open Gastrectomy

Every study compares the short-term outcomes of robotic surgery with the laparoscopic approach in terms of operation time, blood loss, number of harvested lymph nodes and length of hospital stay. Eleven studies [23, 34–38, 40–42, 44, 45] compare the total complication rate after gastrectomy and, of these, three [23, 37, 39] analyse the conversion rate to open technique, five [38, 39, 41, 43, 44] the anastomotic leakage rate, three [38, 39, 41] the anastomotic stenosis rate, two [38, 39] intestinal occlusions, just one [38] the post-operatory bleeding.

Seven [23, 33, 35, 37–39, 43] meta-analyses consider the post-operatory mortality rate, three [33, 39, 43] the morbidity rate.

Only three [34, 35, 39] studies report the time to the first oral intake and five [23, 34, 35, 37, 39] the first time to flatus.

The oncological outcomes in terms of total retrieved lymph nodes were compared in selected meta-analyses. Interestingly, nine [23, 35–39, 41, 42, 44] studies report the proximal and distal margin of resection. Only one meta-analysis [37] reports the 3-year overall survival and the 3-year disease-free survival and another one [35] reports the recurrence free survival.

### Summary of outcomes

In the following paragraph, we describe the findings from the included meta-analyses. In each review we found data for the primary outcomes: operation time, intraoperative bleeding, length of stay and number of harvested lymph-nodes. Along the way, we also analysed other outcomes findings in the selected studies as conversion rate, mortality rate, morbidity, total complication rate, anastomotic leakage, anastomotic stenosis, intestinal obstruction, proximal and distal margin, time to first flatus and for oral intake.

For each review we extracted, for continuous variables, the weighted mean difference (WMD), the 95% confidence interval (95% CI) and the heterogeneity. For the discrete variables, we reported odd ratio (OR), the 95% confidence interval and the heterogeneity (Table 4).

**Table 4:** Outcomes

Operation time (minutes)									
	*N*	N. of patients	RG	LG	WMD	95%CI		*p* value	*I*^2^ (%)
[33]	3	918	268	650	68.77	35.09	102.45	*P* < 0.0001	85
[38]	6	2132	723	1409	50	30.07	69.93	*P* = 0.00	88
[39]	7	2242	667	1575	48.64	29.79	67.5	*P* < 0.0001	87
[40]	6	1214	373	841	63.7	44.22	83.17	*P* < 0.00001	74
[41]	8	1870	643	1236	61.99	43.12	80.86	< 0.001	85
[42]	7	1048	270	778	48.46	29.49	67.43	*P* = 0.000	86.6
[43]	8	2859	898	1961	57.15	42.26	72.05	*P* < 0.00001	88
[44]	5	1796	551	1245	42.9	2087	64.92	*P* = 0.0001	82
[36]	3	562	165	397	21.49	12.48	30.5	*P* < 0.00001	57
[23]	12	3580	1096	2484	42.437	31.82	53.053	*P* < 0.0001	89.7
[34]	9	3250	924	2326	53.48	38.84	68.12	*P* = 0.00	87.1
[35]	19	5953	1830	4123	49.05	39.91	58.18	*P* < 0.01	88
[36]	11	3374	949	2425	42	28.11	55.89	*P* < 0.00001	88
[37]	16	4586	1517	3069	57.98	42.96	73	*P* < 0.00001	94

Blood loss (ML)									
	*N*	n. of patients	RG	LG	WMD	95%CI		*p* value	*I*^2^ (%)
[33]	3	918	268	650	− 41.88	− 71.62	− 12.14	*P* = 0.006	73
[38]	6	2031	687	1344	− 46.97	− 87.83	− 6.12	*P* = 0.02	98
[39]	6	2123	613	1510	− 33.56	− 59.82	− 7.3	*P* = 0.01	93
[40]	5	1113	337	776	− 35.53	− 66.98	− 4.09	*P* = 0.03	75
[41]	8	1870	634	1236	− 6.08	− 25.73	13.58	*P* = 0.54	83
[42]	6	666	170	496	− 38.43	− 67.55	− 9.3	*P* = 0.01	93.3
[43]	7	2758	862	1896	− 28.59	− 56.57	− 0.62	*P* = 0.05	92
[44]	4	1695	515	1180	− 16.07	− 32.78	0.64	*P* = 0.006	75
[36]	3	562	165	397	− 16.6	− 61.31	28.11	*P* = 0.47	94
[23]	11	1028	2416	3444	− 29.855	− 46.236	− 13.474	*P* < 0.0001	94.3
[34]	8	3149	888	2261	− 36.5	− 61.39	− 11.61	*P* = 0.00	92.1
[35]	18	5817	1762	4055	− 24.38	− 36.43	− 12.32	*P* < 0.01	93
[36]	11	3374	949	2425	− 23.68	− 42.25	− 5.1	*P* = 0.01	91
[37]	16	4586	1517	3069	− 23.71	− 40.1	− 7.32	*P* = 0.005	89

Harvested lymphnodes									
	*N*	n. of patients	RG	LG	WMD	95%CI		*p* value	*I*^2^ (%)
[33]	3	918	268	650	0.71	− 6.78	5.36	*P* = 0.82	87
[38]	6	1853	662	1191	1.61	− 1.17	4.39	*P* = 0.26	85
[39]	7	1963	606	1357	1.28	− 2.19	4.76	*P* = 0.47	78
[40]	6	1214	373	841	0.5	− 3.3	4.3	*P* = 0.80	80
[41]	8	1870	634	1236	− 0.25	− 3.72	3.22	*P* = 0.89	81
[42]	7	1493	406	1087	1.06	− 2.33	4.45	*P* = 0.54	74.1
[43]	8	2580	837	1743	0.63	− 2.24	− 3.51	*P* = 0.67	78
[44]	5	1796	551	1245	2.45	0.94	3.95	*P* = 0.001	0
[36]	2	200	85	115	− 0.23	− 3.8	3.35	*P* = 0.9	0
[23]	11	2823	951	1872	2.11	0.63	3.59	*P* = 0.005	67.1
[34]	8	2144	627	1517	1.49	− 1.74	4.72	*P* = 0.00	74.3
[35]	17	4814	1585	3229	1.44	− 0.37	3.26	*P* = 0.12	86
[36]	10	2998	849	2143	0.91	− 1.16	2.99	*P* = 0.39	70
[37]	14	3434	1269	2165	1.81	0	3.62	*P* = 0.05	74

Hospital stay (days)									
	*n*	n. of patients	RG	LG	WMD	95%CI		*p* value	*I*^2^ (%)
[33]	3	918	268	650	− 0.54	− 1.87	0.79	*P* = 0.42	63
[38]	5	2011	685	1326	− 0.5	− 1.07	− 0.08	*P* = 0.09	15
[39]	7	2242	667	1575	− 1.16	− 2.42	0.1	*P* < 0.00001	76
[40]	6	1111	334	777	− 0.43	− 1.46	0.61	*P* = 0.42	87
[41]	8	1870	634	1236	− 0.6	− 1.39	0.2	*P* = 014	56
[42]	6	666	170	496	− 0.1	− 2.57	0.56	*P* = 0.209	80.6
[43]	7	2832	882	1950	− 0.16	− 0.87	− 0.55	*P* = 0.65	0
[44]	4	1675	513	1162	− 1.98	− 3.66	− 0.3	*P* = 0.002	81
[36]	3	562	165	397	0.19	− 0.91	1.3	*P* = 0.74	0
[23]	10	3345	1008	2337	− 0.465	− 0.741	− 0.19	*P* = 0.001	20.3
[34]	9	3250	924	2326	− 1.11	− 2.28	0.06	*P* = 0.00	73.1
[35]	19	5953	1830	4123	− 0.35	− 0.95	0.25	*P* = 0.25	82
[36]	11	3374	949	2425	− 0.65	− 1.53	0.23	*P* = 0.15	84
[37]	14	4345	1438	2907	− 0.49	− 0.99	0.02	*P* = 0.06	45

Total complications									
	*n*	n. of patients	RG	LG	OR	95%CI		*p* value	*I*^2^ (%)
[38]	7	2235	762	1473	1.07	0.82	1.4	*P* = 0.61	0
[40]	6	1220	373	847	0.87	0.57	1.28	*P* = 0.69	0
[41]	8	1870	634	1236	1.12	0.83	1.52	*P* = 0.44	0
[42]	8	1875	506	1369	0.95	0.7	1.28	*P* = 0.713	0
[44]	5	1796	551	1245	1.05	0.77	1.44	*P* = 0.76	0
[36]	3	552	165	397	1.37	0.81	2.3	*P* = 0.24	0
[23]	12	3580	1096	2484	1.02	0.78	1.32	*P* = 0.897	0
[34]	11	3503	993	2510	1.02	0.81	1.27	*P* = 0.8	0
[35]	19	5953	1830	4123	1.05	0.88	1.26	*P* = 0.56	0
[36]	12	3744	1134	2610	1.12	0.89	1.41	*P* = 0.33	0
[37]	14	4426	1487	2939	1.05	0.86	1.28	*P* = 0.65	2

Proximal margins (CM)									
	*n*	n. of patients	RG	LG	WMD	95%CI		*p* value	*I*^2^ (%)
[38]	3	389	171	218	0.31	0.18	0.8	*P* = 0.22	0
[39]	3	1519	510	1009	0.11	− 0.17	0.39	*P* = 0.42	0
[41]	5	1674	561	1113	− 0.06	− 0.32	0.19	*P* = 0.10	49
[42]	3	1049	310	739	0.1	− 0.25	0.45	*P* = 0.56	0
[44]	3	1519	510	1009	0.11	− 0.17	0.4	*P* = 0.42	0
[23]	5	1067	374	693	− 0.104	− 0.307	0.099	*P* = 0.314	0
[35]	9	3030	2006	1024	0.14	− 0.07	0.36	*P* = 0.18	26
[36]	5	2456	723	1733	0.1	− 0.08	0.28	*P* = 0.26	4
[37]	6	615	N.D	N.D	0.34	− 0.12	0.81	*P* = 0.15	0

Distal margins (CM)									
	*n*	n. of patients	RG	LG	WMD	95%CI		*p* value	*I*^2^ (%)
[38]	3	389	171	218	− 0.28	− 0.8	− 0.23	*P* = 0.28	0
[39]	3	1519	510	1009	1.13	0.67	1.6	*P* < 0.00001	0
[41]	5	1674	561	1113	− 1.14	− 1.55	− 0.72	*P* < 0.01	0
[42]	3	1049	310	739	1.04	0.46	1.62	*P* = 0.001	0
[44]	3	1519	510	1009	1.13	0.67	1.6	*P* < 0.00001	0
[23]	5	1067	374	693	− 0.176	− 0.413	0.062	*P* = 0.147	0.278
[35]	8	2921	973	1948	− 0.09	− 0.65	0.46	*P* = 0.74	81
[36]	10	2456	723	1733	0.18	− 0.67	1.03	*P* = 0.68	68
[37]	5	502	N.D	N.D	0.73	− 0.47	1.93	*P* = 0.23	64

Mortality									
	*n*	n. of patients	RG	LG	OR	95%CI		*p* value	*I*^2^ (%)
[33]	3	918	268	650	1.8	0.3	10.98	*P* = 0.52	0
[38]	3	1464	491	973	1.59	0.42	5.94	*P* = 0.49	0
[39]	8	2374	698	1676	1.66	0.44	6.24	*P* = 0.45	0
[43]	5	2730	849	1881	1.36	0.38	4.88	*P* = 0.63	0
[23]	3	1555	389	1166	0.61	0.12	3.1	*P* = 0.9	0
[35]	7	2951	838	2131	1.62	0.62	4.2	*P* = 0.32	0
[37]	14	2895	808	2087	1.35	0.6	4.14	*P* = 0.56	0

Morbidity									
	*n*	n. of patients	RG	LG	OR	95%CI		*p* value	*I*^2^ (%)
[33]	3	918	268	650	0.74	0.47	1.16	*P* = 0.19	0
[39]	9	2495	736	1759	1.13	0.86	1.47	*P* = 0.38	0
[43]	5	2730	849	1881	1.36	0.38	4.88	*P* = 0.63	0

Anastomotic leakage									
	*n*	n. of patients	RG	LG	OR	95%CI		*p* value	*I*^2^ (%)
[38]	6	2171	746	1425	1.61	0.92	2.64	*P* = 0.05	0
[39]	8	2245	711	1534	1.06	0.6	1.89	*P* = 0.83	0
[41]	8	1870	634	1236	1.06	0.57	1.94	*P* = 0.86	0
[43]	5	2730	849	1881	1.16	0.68	1.96	*P* = 0.59	0
[44]	3	1519	510	1009	0.98	0.51	1.9	*P* = 0.95	0

Anastomotic stenosis									
	*n*	n. of patients	RG	LG	OR	95%CI		*p* value	*I*^2^ (%)
[38]	4	874	310	564	0.8	0.24	2.64	*P* = 0.72	0
[39]	7	948	275	673	0.67	0.18	2.5	*P* = 0.55	0
[41]	8	1870	634	1236	0.9	0.29	2.77	*P* = 0.85	0

Intestinal obstruction									
	*n*	n. of patients	RG	LG	OR	95%CI		*p* value	*I*^2^ (%)
[38]	4	1949	672	1277	1.38	0.55	3.46	*P* = 0.48	0
[39]	7	2095	681	1414	1.64	0.43	2.53	*P* = 0.92	0

Time to first flatus (days)									
	*n*	n. of patients	RG	LG	WMD	95%CI		*p* value	*I*^2^ (%)
[39]	2	128	52	76	− 0.44	− 1.15	0.27	*P* = 0.22	0
[23]	5	1174	444	730	− 0.26	− 0.38	− 0.14	*P* < 0.0001	0
[34]	2	134	58	76	− 0.44	− 1.15	0.27	*P* = 0.78	0
[35]	9	1944	713	1231	− 0.09	− 0.27	0.1	*P* = 0.36	74
[37]	7	1045			− 0.2	− 0.42	− 0.02	*P* = 0.07	53

Oral intake (days)									
	*n*	n. of patients	RG	LG	WMD	95%CI		*p* value	*I*^2^ (%)
[39]	3	1611	477	1134	− 0.28	− 0.46	− 0.09	*P* = 0.004	0
[34]	3	1611	477	1134	− 0.28	− 0.46	− 0.09	*P* < 0.01	0
[35]	9	3151	2055	1096	− 0.23	− 0.34	− 0.13	*P* < 0.0001	0

Conversion rate									
	*n*	n. of patients	RG	LG	OR	95%CI		*p* value	*I*^2^ (%)
[39]	6	994	225	769	2.14	0.32	14.14	*P* = 0.43	
[23]	4	1174	314	920	1.55	0.6	4.02	*P* = 0.36	17
[37]	4	1231	365	866	1.58	0.6	4.14	*P* = 0.35	0

Green are the meta-analyses that compares RG to LG and to Open Gastrectomy

### Primary outcomes

#### Operative time

All selected meta-analyses report higher operative time for the robotic group compared to laparoscopy. A statistically significance reduction is reported in eleven studies [23, 33, 35–37, 39–41, 43–45]. It was an unsurprising result due to extra time for robotic docking and undocking as reported in literature [20].

#### Intraoperative bleeding

All studies report a reduction of intraoperative bleeding in the group of robotic gastrectomy compared to laparoscopic gastrectomy and only in three there is not a statistical significance [34, 41, 45]. This result could be explained with the operatory field magnification obtained with robotic three-dimensional optic, associated with the higher precision in the small movements and the flapping filters of robotic system [46].

#### Length of hospital stay

Each meta-analysis report a small reduction in terms of hospital stay for patients who underwent robotic gastrectomy, except for Wang Z et al. [45] that report a negligible increase in hospitalization for RG groups. These results are strengthened by statistically significance only in three meta-analyses [23, 39, 44].

#### Number of harvested lymph nodes

The number of harvested lymph nodes is a significant value for the oncological outcomes of gastrectomy: in 12 meta-analyses [23, 33–38, 40, 42–44] this number is higher for robotic technique and only in two studies [41, 45] is lower than laparoscopic gastrectomy. Only two meta-analyses show statistical significative results [23, 44] and report a higher number of harvested lymph nodes.

### Secondary outcomes

#### Overall complication rate

Eleven meta-analyses [23, 34–38, 40–42, 44, 45] reported the incidence of overall complication after surgery and no significant difference between robotic and laparoscopic gastrectomy in terms of incidence of total complications was reported.

#### Proximal and distal margin of resection

Nine studies [23, 36–40, 42, 43, 45] analysed the difference between the length of proximal and distal margin of resection from the tumour: the proximal margin was more distant in patients with RG in seven studies[35–39, 42, 44], in only one [41] the distance was substantially the same and in another one [23] it was higher in patients who underwent LG. Overall, these data does not show statistical significance. The distal margin was more distant in RG in five studies [36, 37, 39, 42, 44], in four [23, 35, 38, 41] the distance was higher in the LG: three studies [39, 42, 44] with statistical significance report an increased length of free distal margin of resection and only one [41] a reduction of this parameter.

#### Mortality and morbidity

Six meta-analyses [33, 35, 37–39, 43] report higher mortality rate in RG, while Hu LD et al. [23] report lower mortality, with no statistical significance. Only three studies report the morbidity rate after gastrectomy with conflicting results. Xiong et al. [33] outlined a lower morbidity in RG, while others [39, 43] found opposite results, with no statistical significance either way.

#### Anastomotic leakage, anastomotic stenosis and intestinal obstruction

Five studies report anastomotic leak rate: four papers [38, 39, 41, 43] indicate a higher incidence of anastomotic leakage in patients who underwent RG and one [44] a lower incidence. Three meta-analyses [38, 39, 41] outline a lower incidence of anastomotic stenosis and two [38, 39] a higher recurrence of intestinal occlusion. All these findings are not significant from a statistical point of view.

#### Time to first flatus and oral intake

Five meta-analyses [23, 34, 35, 37, 39] investigate the time to first flatus and only three [34, 35, 39] the time for the first oral intake after surgery: all the studies indicate a shorter recovery of bowel function in patient underwent to RG and, of these, one [23] is statistically significant for time to flatus and all [34, 35, 39] are such for time to oral intake.

#### Conversion to open surgery

The risk of conversion to open surgery is higher in RG for all three studies [23, 37, 39] that investigate this issue. Nevertheless no statistical significance is reported.

#### Stratification of evidence

Based on the previously reported classification method we obtained three different levels of evidence for each outcome analysed in the review: only the oral intake is supported by suggestive evidence, operation time and blood loss are supported by weak evidence and the other outcomes are classified as non-significant (Table 5).

**Table 5:** Stratification of evidence

	Sample size (number of cases)	Significance threshold reached (under the random-effects model)	95% prediction interval rule	Estimate of heterogeneity	Small-study effects or excess significance bias	Random-effects summary effect size (95% CI)
	Results supported by suggestive evidence					
Oral intake[34]	> 1000	< 0.001	Including the null value	not large	Neither	0.39 (0.23/0.61)
	Results supported by weak evidence					
Operation time[34]	> 1000	< 0.05 but > 0.001	Including the null value	very large	Neither	0.88 (0.72/1.05)
Blood loss[34]	> 1000	< 0.05 but > 0.001	Including the null value	very large	Neither	0.44(0.65/2.41)
	Non significant Results					
Harvested limphonodes[34]	> 1000	> 0.05	Including the null value	very large	Neither	2.60 (0.66/5.90)
Hospital Stay[34]	> 1000	> 0.05	Including the null value	very large	Neither	0.63 (0.45/0.95)
Total complications[34]	> 1000	> 0.05	Including the null value	not large	Neither	1.05 (0.88/1.26)
Prossimal Margins[34]	> 1000	> 0.05	Including the null value	not large	Neither	0.25 (0.12/0.65)
Distal Margins[34]	> 1000	> 0.05	Including the null value	very large	Neither	0.16 (0.10/0.83)
Mortality[34]	> 1000	> 0.05	Including the null value	not large	Neither	1.62 (0.62/4.2)
Morbidity[42]	> 1000	> 0.05	Including the null value	not large	Neither	1.36 (0.38/4.88)
Anastomotic Leakage[42]	> 1000	> 0.05	Including the null value	not large	Neither	1.16 (0.68/1.96)
Anastomotic Stenosis[40]	> 1000	> 0.05	Including the null value	not large	Neither	0.9 (0.29/2.77)
Intestinal Obstruction[38]	> 1000	> 0.05	Including the null value	not large	Neither	1.64 (0.43/2.53)
Time to first flatus[34]	> 1000	> 0.05	Including the null value	very large	Neither	0.16 (0.03/0.18)
Conversion rate[36]	> 1000	> 0.05	Including the null value	not large	Neither	1.58 (0.6/4.14)

## Discussion

### Main findings and interpretation in light of existing evidence

In this umbrella review of systematic reviews and meta-analyses evaluating the current evidence for potential benefits and harm associated with robotic gastrectomy compared to laparoscopic gastrectomy for gastric cancer, we summarized 14 studies covering overall 146 primary studies, and more than 37,500 subjects. Our assessment did not show an overall excess of findings with significant results, by contrast with other medical specialties, in which an excess of significant results is reported [47–49]. In our study, a large proportion of the examined meta-analyses had a not large heterogeneity and some studies had a large heterogeneity.

The applied Egger test is particularly difficult to interpret when between-study heterogeneity is large. Heterogeneity might often be a manifestation of bias in some studies of a meta-analysis, but could also emerges from genuine differences across studies. Some reasons for heterogeneity include the mixture of cohort studies and case–control studies in some of the meta-analyses, differences in the populations analyzed, in the reproducibility of the surgical technique and in the stage of gastric cancer.

The outcomes reported for each surgical technique need to be interpreted with caution, in particular for the meta-analyses in which the heterogeneity is large, the number of studies is relatively small, the largest study is more conservative than the summary effect.

According to statistical data analyses, robotic gastrectomy is associated with shorter time to oral intake with a high level of evidence. The data regarding lesser intraoperative bleeding and longer operation time for robotic approach are supported by weak evidence. On the other hand, the data regarding other outcomes are insufficient as well as non-significant, from an evidence point of view, to draw any robust conclusion.

As observed in each selected meta-analysis, intraoperative blood loss was significantly lesser in the RG than in the LG groups. From a theoretical point of view, robotic procedure is a more precise technique that could help surgeons visualize small vessels. Furthermore, the robotic arms are more stable than a surgeon's hands, leading to a significant reduction of musculoskeletal fatigue and physiologic tremor over time in surgeons [36]. In addition, the improved dexterity of an internal articulated wrist provides greater flexibility in a restricted operative field, and the stereoscopic vision enables surgeons to effectively minimize the risk of tissue and blood vessel injuries and intraoperative bleeding as well. In the end, we found strong evidence for intraoperative bleeding, shedding light on this benefit of robotic gastrectomy when compared with LG.

Thirteen studies showed that the hospital stay in the RG groups was negligibly shorter (nearly a day) than that in the LG groups, reaching statistical significance in only three meta-analyses. Similarly, other potential factors that should have an important impact on postoperative recovery, such as time to diet and first flatus resulted shorter in RG groups. Based on these results, we postulate that the faster recovery of patients receiving robotic approach induced the different postoperative hospital stay between the 2 groups. The evidence from our study is highly suggestive for these benefits; therefore, surgeons and policy makers should consider the robotic approach as an acceptable option in treating gastric cancer.

As prognostic factors of surgical therapy from an oncological point of view, the number of resected lymph nodes as well as the length of resection margins cannot be ignored. In our umbrella review, even if the number of retrieved nodes obtained with the robotic gastrectomy was higher, the significance was negatively affected by the low value of evidence stratification. Several studies [23, 33–38, 40, 42–44] report that the number of retrieved lymph nodes during extra-perigastric lymphadenectomy, especially in the case of splenic pedicle and splenic hilum and in the supra-pancreatic areas, was significantly higher for the robotic group compared to the laparoscopy group. However, it appears clear that the operative steps of lymph node dissection robotically performed are generally the same as those in laparoscopy. We could postulate that the traditional straight laparoscopic instruments fail to help surgeons reach deep-seated vessels and such nodal areas. In addition, the tremor filtering, wristed instruments, as well as stable exposure and high-resolution image enable surgeons to execute thoroughly surgical maneuvres thoroughly [50]. However, most of the primary studies included patients who underwent both subtotal gastrectomy and total gastrectomy without distinction, and the stage of gastric cancer was also not the same for all of the enrolled patients. Anyway, since case-matching studies according to the type of gastrectomy and the extension of lymphadenectomy comparing robotic and laparoscopic approach are needed to reduce the bias, given that only two out of 12 meta-analyses reached statistical significance, surgeons and policy makers should cautiously consider the marginal superiority of robotic gastrectomy in lymph-node retrieval. In addition, evidence for difference in margins between robotic and laparoscopic groups is only suggestive. As a pathological parameter, the proximal margin was longer in the RG group, while distal margin resulted in controversial results between the two groups. These findings may open up new research directions.

The prolonged operating time in RG was shown in all the included meta-analyses, preluding a negative impact on postoperative outcomes due to prolonged exposure time to pneumoperitoneum and the associated increased anesthesia time. However, previous studies investigating the effect of longer operation time in patients receiving laparoscopic gastrectomy did not show detrimental surgical results [35]. One of the most important reasons of prolonged time is that robotic gastrectomy requires “setting and docking” time for the robot, which inevitably results in a longer operative time requiring almost 30 min of extra time [51–54]. In addition, the learning curve for robotic gastrectomy significantly affect the time spent during surgery. Eom BW et al. [21] stated that intervention time was reduced after at least 15 robotic gastrectomies. On the same way, Woo JH et al. [24] demonstrated a reduction of the mean operative time from 233 to 219 min after the execution of 100 cases. Anyway, considering the development of the robotic surgery systems, more experience, and a shortened learning curve, we can postulate that RG is technically feasible in regard to operation time.

Interestingly, the prolonged operation time of RG was not associated to any increase in postoperative complications, mortality, or conversion rate. It is postulated that technical advantages such as 3D vision and tremor filtering could contribute to safer implementations of the robotic system for gastric surgery [21, 47].

Due to limited meta-analyses included, an umbrella review for cost evaluation was not performed. But Hyun et al. [41] and Chen et al. [35] report that the RG costs an average of €3,189 and 3900 USD, respectively, compared to the LG, of which most of this amount, around €2831 is determined from the DaVinci robotic system itself. On the basis of what was reported by both authors, the possible advantages of the robotic approach would not be justified by the higher cost but looking at the set of costs related to hospitalization, we come to the conclusion that the higher operating costs are finally offset by the reduction of complications and of hospitalization time.

The results from primary studies are consistent with the findings of our umbrella review as we found highly suggestive evidence that RG and LG are equivalent as regard the safety and feasibility, considering the robotic approach as a safe and non-inferior option in treating gastric cancer toward LG.

### Strengths and limitations

We performed this detailed umbrella review to assess the benefits and harm of robotic gastrectomy compared with laparoscopic approach. In addition, we used a comprehensive and systematic criterion to grade evidence levels to rate the strength of these systematic reviews and meta-analyses. Our review inevitably has limitations and drawbacks. First, we fully trust the accuracy of the data provided in the included meta-analyses. As such, problems within the published data may impact the evidence-rating results despite our analyses. All the meta-analyses included in this review compared retrospective non-randomized studies and until now no randomized clinical trials (RCTs) are available between RG and LG. Another limitation is that significant heterogeneity was recognized in some characteristics of the primary studies. Several papers included patients who underwent both subtotal gastrectomy and total gastrectomy without distinction. The stage of gastric cancer was also not the same for all of the enrolled patients. The majority of the studies were from Eastern populations, whereas the minority were from Europe. The classification of evidence supporting the single outcome highlighted how no outcomes analysed in the creation of this review are supported by convincing or highly suggestive evidence.

On the other hand, we are convinced of the strength of our umbrella review, since the methodological quality of all included systematic reviews and meta-analyses were considered critically high.

## Conclusions

In conclusion, the safety and efficacy of robotic gastrectomy are not clearly supported by strong evidence, suggesting that the outcomes reported for each surgical technique need to be interpreted with caution, in particular for the meta-analyses in which the heterogeneity is large. Certainly, robotic gastrectomy is associated with shorter time to oral intake, lesser intraoperative bleeding and longer operation time with an acceptable level of evidence. On the other hand, the data regarding other outcomes are insufficient as well as non-significant, from an evidence point of view, to draw any robust conclusion.

## Appendix 1: PRISMA checklist

**Table:**

Section/topic	#	Checklist item	Reported on page #
Title			
Title	1	Identify the report as a systematic review, meta-analysis, or both	1
Abstract			
Structured summary	2	Provide a structured summary including, as applicable: background; objectives; data sources; study eligibility criteria, participants, and interventions; study appraisal and synthesis methods; results; limitations; conclusions and implications of key findings; systematic review registration number	2
Introduction			
Rationale	3	Describe the rationale for the review in the context of what is already known	3
Objectives	4	Provide an explicit statement of questions being addressed with reference to participants, interventions, comparisons, outcomes, and study design (PICOS)	3
Methods			
Protocol and registration	5	Indicate if a review protocol exists, if and where it can be accessed (e.g., Web address), and, if available, provide registration information including registration number	4
Eligibility criteria	6	Specify study characteristics (e.g., PICOS, length of follow-up) and report characteristics (e.g., years considered, language, publication status) used as criteria for eligibility, giving rationale	Into Protocol
Information sources	7	Describe all information sources (e.g., databases with dates of coverage, contact with study authors to identify additional studies) in the search and date last searched	4
Search	8	Present full electronic search strategy for at least one database, including any limits used, such that it could be repeated	4
Study selection	9	State the process for selecting studies (i.e., screening, eligibility, included in systematic review, and, if applicable, included in the meta-analysis)	4
Data collection process	10	Describe method of data extraction from reports (e.g., piloted forms, independently, in duplicate) and any processes for obtaining and confirming data from investigators	4
Data items	11	List and define all variables for which data were sought (e.g., PICOS, funding sources) and any assumptions and simplifications made	Into Protocol
Risk of bias in individual studies	12	Describe methods used for assessing risk of bias of individual studies (including specification of whether this was done at the study or outcome level), and how this information is to be used in any data synthesis	4
Summary measures	13	State the principal summary measures (e.g., risk ratio, difference in means)	6
Synthesis of results	14	Describe the methods of handling data and combining results of studies, if done, including measures of consistency (e.g., I^2^) for each meta-analysis	6
Risk of bias across studies	15	Specify any assessment of risk of bias that may affect the cumulative evidence (e.g., publication bias, selective reporting within studies)	
Additional analyses	16	Describe methods of additional analyses (e.g., sensitivity or subgroup analyses, meta-regression), if done, indicating which were pre-specified	
Results			
Study selection	17	Give numbers of studies screened, assessed for eligibility, and included in the review, with reasons for exclusions at each stage, ideally with a flow diagram	5
Study characteristics	18	For each study, present characteristics for which data were extracted (e.g., study size, PICOS, follow-up period) and provide the citations	5
Risk of bias within studies	19	Present data on risk of bias of each study and, if available, any outcome level assessment (see item 12)	
Results of individual studies	20	For all outcomes considered (benefits or harms), present, for each study: (a) simple summary data for each intervention group (b) effect estimates and confidence intervals, ideally with a forest plot	6
Synthesis of results	21	Present results of each meta-analysis done, including confidence intervals and measures of consistency	5–6-7
Risk of bias across studies	22	Present results of any assessment of risk of bias across studies (see Item 15)	
Additional analysis	23	Give results of additional analyses, if done (e.g., sensitivity or subgroup analyses, meta-regression [see Item 16])	
Discussion			
Summary of evidence	24	Summarize the main findings including the strength of evidence for each main outcome; consider their relevance to key groups (e.g., healthcare providers, users, and policy makers)	9
Limitations	25	Discuss limitations at study and outcome level (e.g., risk of bias), and at review-level (e.g., incomplete retrieval of identified research, reporting bias)	10
Conclusions	26	Provide a general interpretation of the results in the context of other evidence, and implications for future research	11
Funding			
Funding	27	Describe sources of funding for the systematic review and other support (e.g., supply of data); role of funders for the systematic review	1

*From:* Moher D, Liberati A, Tetzlaff J, Altman DG, The PRISMA Group (2009). Preferred Reporting Items for Systematic Reviews and Meta-Analyses: The PRISMA Statement. PLoS Med 6(7): e1000097. https://doi.org/10.1371/journal.pmed1000097

For more information, visit: www.prisma-statement.org.

## Appendix 3: List of included meta-analyses

See references [23, 33–45].
